# Strategies for optimizing BioNano and Dovetail explored through a second reference quality assembly for the legume model, *Medicago truncatula*

**DOI:** 10.1186/s12864-017-3971-4

**Published:** 2017-08-04

**Authors:** Karen M. Moll, Peng Zhou, Thiruvarangan Ramaraj, Diego Fajardo, Nicholas P. Devitt, Michael J. Sadowsky, Robert M. Stupar, Peter Tiffin, Jason R. Miller, Nevin D. Young, Kevin A. T. Silverstein, Joann Mudge

**Affiliations:** 1 National Center for Genome Resources, 2935 Rodeo Park Drive East, Santa Fe, NM 87505 USA; 2 Montana State University, Center for Biofilm Engineering, Bozeman, MT 59717 USA; 3 Department of Plant Biology, University of Minnesota, Saint Paul, MN USA; 4 Department of Soil, Water & Climate, Plant and Microbial Biology and BioTechnology Institute, University of Minnesota, St. Paul, MN USA; 5 Department of Agronomy and Plant Genetics, University of Minnesota, Saint Paul, MN USA; 6 Department of Plant and Microbial Biology, University of Minnesota, Saint Paul, MN USA; 7 J. Craig Venter Institute, Rockville, MD USA; 8 Minnesota Supercomputing Institute, University of Minnesota, Minneapolis, MN USA

**Keywords:** Genome assembly, Next generation sequencing, BioNano, Dovetail, PacBio, *Medicago truncatula*

## Abstract

**Background:**

Third generation sequencing technologies, with sequencing reads in the tens- of kilo-bases, facilitate genome assembly by spanning ambiguous regions and improving continuity. This has been critical for plant genomes, which are difficult to assemble due to high repeat content, gene family expansions, segmental and tandem duplications, and polyploidy. Recently, high-throughput mapping and scaffolding strategies have further improved continuity. Together, these long-range technologies enable quality draft assemblies of complex genomes in a cost-effective and timely manner.

**Results:**

Here, we present high quality genome assemblies of the model legume plant, *Medicago truncatula* (R108) using PacBio, Dovetail Chicago (hereafter, Dovetail) and BioNano technologies. To test these technologies for plant genome assembly, we generated five assemblies using all possible combinations and ordering of these three technologies in the R108 assembly. While the BioNano and Dovetail joins overlapped, they also showed complementary gains in continuity and join numbers. Both technologies spanned repetitive regions that PacBio alone was unable to bridge. Combining technologies, particularly Dovetail followed by BioNano, resulted in notable improvements compared to Dovetail or BioNano alone. A combination of PacBio, Dovetail, and BioNano was used to generate a high quality draft assembly of R108, a *M. truncatula* accession widely used in studies of functional genomics. As a test for the usefulness of the resulting genome sequence, the new R108 assembly was used to pinpoint breakpoints and characterize flanking sequence of a previously identified translocation between chromosomes 4 and 8, identifying more than 22.7 Mb of novel sequence not present in the earlier A17 reference assembly.

**Conclusions:**

Adding Dovetail followed by BioNano data yielded complementary improvements in continuity over the original PacBio assembly. This strategy proved efficient and cost-effective for developing a quality draft assembly compared to traditional reference assemblies.

**Electronic supplementary material:**

The online version of this article (doi:10.1186/s12864-017-3971-4) contains supplementary material, which is available to authorized users.

## Background

Next generation sequencing technologies such as 454, Illumina, and SOLiD became available in the late 2000s [1, 2]{Margulies, 2005 #113}. These technologies have the advantage of extremely high throughput and much lower cost per sequenced base compared to Sanger sequencing [3–8]. Long read sequencing technologies, such as PacBio and Oxford Nanopore, produce reads in the tens- of kilo-base range, much longer than what was possible even with traditional Sanger technology. However, they also have higher error rates, lower throughput, and higher costs per base compared to the short read technologies. Recently, PacBio throughput and cost per base have improved to the point that de novo plant genome assemblies using only PacBio are possible [9, 10].

Concomitantly, the throughput and cost of long-range scaffolding and mapping technologies that can increase continuity of an assembly have also improved dramatically. Traditional physical maps, dependent on expensive BAC library preparation, have given way to a variety of new technologies, including Opgen, Keygene, BioNano, and Nabsys maps [11–15]. BioNano is a high throughput optical mapping technology that utilizes endonucleases to nick long DNA molecules at the enzyme’s recognition site, incorporating fluorescent nucleotides to obtain sequence-based patterns. The specific patterns are then used to assemble DNA molecules into a larger genome map, which can then be used to direct and improve a de novo genome assembly [16].

Genomic architecture analyses also can be achieved by sequencing libraries produced from chromatin proximity ligation methods such as Hi-C [17]. Dovetail Chicago libraries are similar to Hi-C but rely on library preparation from in vitro rather than in vivo reconstituted chromatin that has been cross-linked and sheared. Dovetail Chicago libraries also use extraction of high molecular weight DNA extraction which limits input DNA length compared to Hi-C, which uses intact chromosomes. These libraries retain proximity signal with sequences physically close together being linked more often than those farther apart. This generates sequence pairs with insert sizes that can be as large as the size of the input DNA, typically ~100 kb, for use in scaffolding with Dovetail’s in-house software [18].

Although BioNano and Dovetail are both long-range scaffolding technologies, there are several important differences. While both rely on restriction endonuclease digestions, different restriction enzymes are used for both technologies, potentially introducing different regional biases. Dovetail and BioNano also differ in the way they handle gaps. Dovetail does not attempt to size the gap, but instead adds 100 Ns between scaffolds that it joins. By contrast, BioNano estimates gap size. Consequently, BioNano can appear to increase scaffold size more when the same scaffolds are joined with both technologies. In addition, BioNano does not automatically split sequences while Dovetail does. BioNano produces a file with possible chimeric sequences, but splitting of these sequences requires manual intervention by the user.

These new sequencing and mapping technologies have increased throughput, driven down costs, and introduced important technological advantages facilitating the sequencing of plant genomes, which are notoriously difficult due to large-scale duplications and repeats [19]. Indeed, these technologies are enabling the construction of multiple high quality plant genome assemblies [4, 6, 7, 9, 20–29] and are now poised to increase the number of sequenced plant genomes even further.

Because legumes (family *Fabaceae*) are important in both agriculture and natural ecosystems, primarily due their capacity to form symbiotic relationships with nitrogen fixing bacteria, multiple genome assemblies are now available. Reference assemblies exist for lotus (*Lotus japonicus*) [30], soybean (*Glycine max*) [31], medicago (*Medicago truncatula*) [32], chickpea (*Cicer arietinum*) [33], mungbean (*Vigna radiata*) [34] and peanut (*Arachis sp.*) [10, 35]. Recently, multiple genome assemblies of a single plant species have begun to appear, enabling the identification of variation in genome content and structure segregating within species [36–40], including legumes [36, 39].

*Medicago truncatula* is a widely studied legume genome, especially in the area of plant-bacterial symbioses. Two *Medicago* accessions have been mainly used for genomic studies, R108 and A17 (Tadege et al. 2008, Young 2011). The relationship of R108 to A17, the accession used for generating the *M. truncatula* reference genome, makes it valuable both for a technology comparison and as a second *M. truncatula* assembly. Genotype R108 is one of the most distant *M. truncatula* accessions from A17 [41]. Relative to A17, R108 has much higher transformation efficiency, has a shorter generation time, and is easier to germinate, making it attractive for genetic studies [42]. Also, R108 is also important to the plant and symbiosis communities because it is the accession that was used to create a large Tnt1-insert population, widely used in functional analysis [42, 43]. Having two high quality references in *Medicago* therefore allowed us to perform comprehensive genome-scale comparisons between the two assemblies, revealing additional novel R108 sequences as well as increased fine-structure details of important re-arrangement events compared to previous analyses using ALLPATHS-LG assemblies [39].

*M. truncatula* has a modest genome size, approximately 465 Mb [44]. However, it also has an evolutionary history of whole genome duplications [45, 46] and frequent local duplications, which appear to be particularly common in this plant species [32], both of which make assembly difficult. We therefore generated and evaluated five combinations of PacBio, BioNano, and Dovetail technology to see how the technologies could complement each other and to explore differences in the ordering of technologies. Ultimately, we present a second, high quality reference genome for *M. truncatula* accession R108, based on an optimized combination of the three sequencing/mapping technologies.

## Results

Assembly Pb was generated using ~100X PacBio coverage and the FALCON assembler followed by Quiver polishing. Four additional assemblies were then created that had either BioNano (PbBn), Dovetail (PbDt), or both scaffolding technologies added onto the base assembly. The assemblies with both scaffolding technologies were created by applying BioNano and then Dovetail (PbBnDt) or Dovetail and then BioNano (PbDtBn).

### Assembly continuity

The Pb base assembly had just over 1000 contigs with no gaps in the sequence (Table 1). It totals just under 400 Mb compared to 412 Mb assembled in the *M. truncatula* A17 reference out of the estimated 465 Mb genome size. The contig N50 for the Pb assembly is 3.77 Mb and the longest sequence is 13.59 Mb. We then added mapping or scaffolding technologies (BioNano and/or Dovetail) on top of this base assembly to improve scaffolding.

**Table 1:** Number and characteristics of contigs and scaffolds for each of the five assemblies

	PacBio (Pb)	PacBio BioNano (PbBn)	PacBio Dovetail (PbDt)	PacBio BioNano Dovetail (PbBnDt)	PacBio Dovetail BioNano (PbDtBn)
Assembly software	FALCON	FALCON Irys	FALCON HiRise	FALCON Irys HiRise	FALCON HiRise Irys
Contigs	1, 073	1, 073	1, 121	1, 125	1, 121
Contig Length	396,973,838	396,973,942	396,973,838	396,973,942	396,973,934
Contig N50^a^	3,768,504	3,768,512	3,768,504	3,768,512	3,768,504
Scaffolds	1, 073	993	1, 005	965	942
Scaffold Length	396,973,838	401,421,527	396,985,438	401,429,527	399,955,467
Maximum Scaffold Length	13,488,151	22,885,216	19,275,758	12,137,306	12,557,854
Scaffold N50^a^	3,768,504	6,819,834	6,895,511	12,137,306	12,557,854

^a^N50 s were also adjusted to use an assembly length of 400 Mb for all assemblies in order to facilitate comparisons across assemblies. Scaffold and contig N50 s adjusted for a 400 Mb assembly size were identical to unadjusted N50 s shown above, except for the PbDt scaffold N50 for which the adjusted N50 was 6,348,449 nt

Both BioNano and Dovetail (PbBn or PbDt) technologies improved the PacBio only base assembly in similar ways (Table 1). The number of scaffolds decreased in both assemblies, dropping by 80 scaffolds in the PbBn assembly and 68 scaffolds in the PbDt assembly while having little effect on total scaffold length (Table 1). The PbBn assembly increased the scaffold length by approximately 1%, adding 4.4 Mb, likely reflecting the fact that BioNano, unlike Dovetail, sizes the gaps it makes when joining sequences. Dovetail adds 100 Ns for each gap it creates, adding only 11.6 kb to the scaffold length.

The scaffold N50 s increased substantially for both the PbBn and PbDt assemblies, from 3.8 Mb in the base Pb assembly to over 6.8 Mb in both assemblies (Table 1). Although the scaffold N50 was slightly higher in the PbDt assembly (6.9 Mb vs 6.8 Mb), the N50 when adjusted for total genome size to allow for comparisons across assemblies (adjusted N50) dropped to 6.3 Mb in the PbDt assembly but remained unchanged in the PbBn assembly. Maximum scaffold sizes increased in both assemblies, from 13.5 Mb in the Pb assembly to 22.1 Mb in the PbBn assembly and 19.3 Mb in the PbDt assembly.

Adding a second technology to the PbBn and PbDt assemblies resulted in two assemblies that differed only in the order in which the BioNano and Dovetail technologies were applied. Overall, the PbBnDt and PbDtBn assemblies were very similar by scaffold size metrics (Table 1). Combining all three technologies resulted in slight decreases in the number of scaffolds, slight increases in total scaffold length, and large increases in scaffold N50 (Table 1). The increase in continuity was particularly striking, with the scaffold N50 nearly doubling to over 12 Mb relative to the PbBn and PbDt assemblies and nearly tripling relative to the Pb base assembly. The maximum scaffold length was slightly larger in the PbBnDt assembly (30.4 Mb vs 27.3 Mb in the PbDtBn assembly), though the PbDtBn assembly had a slightly larger increase over its input assembly (PbDt).

As expected, given that neither BioNano nor Dovetail added a significant amount of sequence data, the number of contigs, contig lengths, and N50 s, were nearly identical for all five assemblies (Table 1). The only substantial change to the contig stats was a slight increase in the number of contigs when Dovetail technology was used, due to the breaking of chimeric contigs (Table 1).

### Assembly completeness

To assess assembly completeness we examined the number of genomic reads that were captured by the assembly. We used PacBio reads, which were used to create the assemblies, as well as Illumina reads, which represent an independent read set, that were captured by the assemblies. The base (Pb) assembly captured 91.8% of the PacBio reads and 96.8% of the Illumina reads. Moreover, 95.7% of the Illumina reads aligned as pairs with expected orientation and distance, indicating that, at least on the local scale, the assembly is accurate.

Because BioNano and Dovetail are scaffolding technologies, they are not expected to add a substantial amount of additional sequence, but rather to organize the assembly sequences into longer scaffolds. Indeed, the estimates of assembly completeness obtained through read capture did not change meaningfully upon the addition of these technologies (Additional file 1: Table S1).

### Gene space completeness

In order to investigate the completeness of the gene space in the five assemblies we determined rates of capture for conserved single-copy eukaryotic genes (BUSCO) [47] and an R108 transcriptome assembly, and assessed MAKER-P annotations. Because completeness results for all 5 assemblies were quite similar we discuss only results for the Pb base assembly and present results for the other assemblies in the supplement (Additional file 1: Table S2). The BUSCO analysis indicates that the base assembly (Pb) captured nearly all of the genes (878 of the 956 genes in the dataset; 91.8%). Nearly 16% (151) of the putative single-copy genes in the BUSCO database were duplicated within the assemblies. These putative duplicates might be due to true duplications in the R108 genome or they might be due to artificial redundancy in the assembly. Even though the BUSCO gene groups are generally single copy, given plant genome duplication rates it isn’t surprising that some of the genes are duplicated.

In addition to looking at capture of conserved genes, we also looked at capture of an R108 RNA-Seq assembly that was produced independently of the genome. Assembly completeness results were similar to those seen with BUSCO, with approximately 92% (94,519) transcripts captured. However, as would be expected, the duplication rate was much higher than that seen in BUSCO, which specifically focuses on single copy genes. In the R108 transcript assembly, 37,929 transcripts (37% of total, 40.1% of aligned transcripts) were duplicated.

Finally, we analyzed the total number of genes predicted from MAKER-P. There were 54,111 genes compared to 50,894 gene loci in Mt4.0 (accession A17). This gives additional confirmation that the gene space is largely complete. Further, there may be additional genes in the R108 Pb assembly not found in A17 (see below).

### Joins and breaks

When characterizing the joins made by BioNano and Dovetail, some interesting trends emerged (Additional file 1: Table S3). Dovetail joined more scaffolds when applied to the base (Pb) assembly compared to BioNano. Dovetail joined 172 Pb scaffolds into 64 PbDt scaffolds while BioNano joined 140 Pb scaffolds into 50 PbBn scaffolds. The same trend of more joins for Dovetail compared to BioNano held when adding a second scaffolding or mapping technology. Dovetail joined 114 PbBn scaffolds into 45 PbBnDt scaffolds and BioNano joined 96 PbDt scaffolds into 33 PbDtBn scaffolds. For the two contrasting assemblies created with all technologies, the two rounds of scaffolding resulted in a total of 254 scaffolds joined in the PbBnDt assembly and 268 scaffolds joined in the PbDtBn assembly, a difference of just over 5%. While Dovetail joined more scaffolds, BioNano had a higher average number of scaffolds per join (Additional file 1: Table S3).

To determine the characteristics of scaffolds that were being joined, we pulled out scaffolds from the input assembly that were joined by either technology in either round (Table 2, Additional file 1: Table S4). The biggest difference between the two technologies was in the ability to join shorter scaffolds. Dovetail was able to join scaffolds as short as 4765 nucleotides into a larger super-scaffold (in both rounds 1 and 2), whereas the minimum scaffold size that BioNano was able to join was 172,295 in round 1 and 98,093 in round 2. To further understand the ability of Dovetail to join smaller contigs, we quantified the number of input scaffolds less than 100 kb that each technology was able to join (Additional file 1: Table S4). Dovetail joined 35 sub-100 kb scaffolds (17 in round 1 and 18 in round 2). BioNano, on the other hand joined only 1 sub-100 kb scaffold total (in round 2), and that scaffold was nearly 100 kb (98,093 nt). Clearly, Dovetail is better at incorporating short scaffolds less than 100 kb.

**Table 2:** Characteristics of input scaffolds that were joined by BioNano and/or Dovetail

Assembly	Pb - > PbDt	Pb - > PbBn	PbDt - > PbDtBn	PbBn - > PbBnDt
Scaffolds	172	140	96	114
Max Scaffold	13,488,151	13,488,151	19,275,758	22,885,216
Scaffold N50	3,957,684	3,698,567	6,895,511	6,819,834
Scaffold N90	854,372	929,179	1,425,957	1,427,073
Min Scaffold	4, 765	172,295	98,093	4, 765
Total Scaffold Length	307,402,024	293,002,927	260,974,793	289,680,947

While Dovetail appears to be better at incorporating shorter scaffolds, it also appears to more effectively join longer scaffolds. When only scaffolds > = 100 kb cutoff were examined, Dovetail joined 253 input scaffolds and BioNano joined 237 across both rounds. Similarly, when only very large scaffolds were examined (> = 1 Mb) Dovetail joined 141 input scaffolds and BioNano joined 128 across both rounds. Dovetail had a higher number of joins at each cutoff when the data were broken down by each round as well (data not shown).

To identify similarities between the two technologies, we determined whether some of the joins made were the same between BioNano and Dovetail. We focused on the first round, where each technology was added onto the Pb assembly, looking for cases where the same Pb scaffolds were joined into a super-scaffold. There were 47 Pb input scaffolds that were scaffolded by both BioNano and Dovetail, resulting in 21 scaffolds in the PbDt assembly and 20 scaffolds in the PbBn assembly. The fact that these joins were made by two independent technologies improves our confidence in these joins. Given that there were also joins made that were unique to both technologies supports the increased continuity and additional joins that we are seeing in assemblies that have both technologies added.

In order to determine whether Dovetail was breaking apart scaffolds that BioNano had previously created by merging Pb scaffolds, we looked further into the Dovetail breaks. In other words, we asked whether any of the joins made by BioNano when generating the PbBn assembly were subsequently split by Dovetail when applied to the PbBn assembly to generate the PbBnDt assembly. From the merged scaffolds generated in the PbBn assembly, only 8 PbBn scaffolds were broken by Dovetail in the PbBnDt assembly and no breaks occurred directly inside the gaps that had been generated by BioNano (median distance from gap was 137,686 nt). We generally found read support spanning these regions, with half or more of the alignments having equally good hits to other regions of the assembly (data not shown). This indicates that these were large repetitive regions and it was difficult to say confidently whether the region should be joined (BioNano correct) or broken (Dovetail correct).

### Joins and breaks in relation to A17

We used alignments of first round assembly scaffolds (PbBn and PbDt) to A17 to predict whether scaffold joins were correct. If joined pieces of a scaffolds mapped to the same A17 chromosome, this lends support for the join. Because of the evolutionary distance between R108 and A17, rearrangements are expected, so a negative result doesn’t necessarily mean the join is incorrect. However, vastly different rates of A17 synteny between scaffold joins made by BioNano and Dovetail would suggest better accuracy for one of the technologies.

Scaffolds joined by BioNano mapped to the same A17 chromosome at a rate of 78.57% while those joined by Dovetail mapped to the same A17 chromosome at a rate of 93.75%. This suggests that Dovetail had a better accuracy than BioNano. Scaffolds with joins that were supported by both BioNano and Dovetail appear to be of higher accuracy based on alignments to A17. For BioNano, while over half of joins (54.54%) were from scaffolds that had similar joins by Dovetail, only 20.00% of joins that mapped to different A17 chromosomes were supported by a similar Dovetail scaffold. This resulted in a 90.91% of Dovetail-supported BioNano joins that mapped to the same A17 chromosome, an increase of 12.34% over all BioNano joins. Dovetail, had more joins than BioNano (see above), with 36.67% of the joins supported by a similar BioNano scaffold. A similar percentage was seen in the number of BioNano-supported Dovetail joins compared to all Dovetail joins (33.33%), resulting in 94.29% of BioNano-supported Dovetail joins aligning to a single A17 chromosome, representing an increase of 0.54%.

Finally, we looked at A17 synteny in the eight PbBn scaffolds that were subsequently broken by Dovetail in the PbBnDt assembly. Three of the scaffolds had input pieces that mapped to chromosome U (unknown), making it difficult to determine A17 synteny and indicating that repetitive sequence is likely that made it difficult to make a chromosome assignment. Of the other 5 scaffolds, 3 mapped to the same A17 chromosome, supporting the BioNano join and 2 mapped to different chromosomes, supporting the subsequent Dovetail break.

### Gaps

The sizing of gaps in BioNano versus the addition of 100 nts in Dovetail, resulted in an increase in the amount of nucleotides added to the total scaffold length in the first round for BioNano compared to Dovetail (Table 1).

In order to see how the gap strategies of BioNano and Dovetail interact, we analyzed the second round assemblies (PbBnDt and PbDtBn), which have both technologies incorporated but with differing order. When a second scaffolding or mapping technology was added to an assembly that already incorporated the other technology, the gaps from the first technology were carried over intact. As noted above, Dovetail sometimes broke apart scaffolds that BioNano had put together. However, when breaking these scaffolds, Dovetail never broke the scaffolds within the gap generated by BioNano but rather broke it in a nearby position. In assemblies where BioNano was added to the PbDt assembly, the minimum gap size that BioNano introduced was 500 nt. This minimum size might be because 500 nt is the minimum gap BioNano can span. Alternatively, given that the assemblies are all based upon PacBio data, it may be that smaller gaps were easily bridged by the PacBio data itself.

The assemblies with both BioNano and Dovetail (PbBnDt and PbDtBn) ended up with a similar number of captured gaps (Table 3). The maximum gap length was over 647 kb, generated when adding BioNano onto the Pb assembly. Although Dovetail doesn’t size its gaps, given the insert size of ~100 kb, it is likely that most of the gaps fall below this range. BioNano, with a gap N50 of 171,515 (Table 3), therefore was able to jump across larger distances than Dovetail.

**Table 3:** Characteristics of the gaps introduced into the assemblies by BioNano and Dovetail. Note, there are no gaps in the Pb only base assembly so it is not included

	PbBn	PbDt	PbBnDt	PbDtBn
Captured Gaps	80	116	160	179
Max Gap	647,836	100	647,836	647,022
Min Gap	500	100	100	100
Mean Gap	55,595	100	27,847	16,657
Gap N50	171,515	100	171,515	105,896
Total Gap Length	4,447,585	11,600	4,455,585	2,981,533

A similarly sized gap generated when adding BioNano onto the PbDt assembly traces back to the same Pb scaffolds as the join made by BioNano on the Pb assembly. Finally, the total gap length varies. Among those assemblies that contain sized gaps (PbBn, PbBnDt, and PbDtBn), the PbDtBn assembly has considerably fewer nts in gaps compared to the other two. This is somewhat surprising given the fact that this assembly has the most gaps of any assembly and that there were more joins made over the two rounds in the PbDtBn assembly (268) than over both rounds in the PbBnDt assembly (254) (Additional file 1: Table S3). Overall, the gap sizes in PbDtBn are smaller (Table 3), accounting for the lower number of nts in gaps.

Finally, in order to surmise the nature of sequence in the gaps and why contigs stop instead of continuing on, we looked at the sequence flanking the gaps (10 kb). Interestingly, the joins made by BioNano and Dovetail (and the breaks made by Dovetail) were enriched for repetitive sequence in the regions flanking the gap introduced with the join (Additional file 1: Figure S1). BioNano and Dovetail both appear to be able to jump across larger repetitive regions than is possible with PacBio reads. In other words, the value of the two technologies is often in their ability to bridge across repetitive regions that PacBio reads cannot currently cross.

### Ordering of technologies

The ordering of the scaffolding or mapping technologies made a difference to the continuity and completeness statistics (Table 1, Additional file 1: Tables S1 and S2). Using Dovetail before BioNano provides multiple benefits. The fact that Dovetail breaks chimeric scaffolds automatically means that using it up front provides a cleaner assembly template for BioNano. Dovetail’s ability to scaffold much smaller pieces of DNA compared to BioNano means that if Dovetail is used up front, more joins will be made and a better base sequence assembly constructed.

### Final assembly draft

In order to create the best reference assembly, we gap-filled the PbDtBn assembly using PBJelly (named R108 version 1.0, Table 4). The PbDtBn assembly was chosen because it had slightly better assembly stats compared to PbBnDt (Table 1, Additional file 1: Tables S1 and S2). For the five preliminary assemblies interrogated above, we did not do any gap filling or polishing (except that the base assembly was polished with Quiver) because these methods would obscure the effects that the BioNano and Dovetail technologies were having on the assembly process. Nevertheless, PBJelly was used for gap-filling as well as super-scaffolding on the final assembly draft in order to improve continuity. While gap filling can be over-aggressive especially if flanking sequences are repetitive, having some sequence, even if not perfect, is often better than having just Ns. In addition, using Dovetail and then BioNano enabled us to use independent data to bring scaffolds together and size the gap between them, making us more confident with doing gap-filling.

**Table 4:** Assembly Statistics for R108 version 1.0 (PbDtBn PBJelly gap filled) and its input assembly (PbDtBn)

	R108 v 1.0	PbDtBn
Contigs	1, 016	1, 121
Contig Length	399,348,944	396,973,934
Contig N50	5,925,378	3,768,504
Scaffolds	909	942
Scaffold Length	402,065,285	399,955,467
Scaffold N50	12,848,239	12,557,854

PBJelly was able to fill many of the captured gaps, increasing the continuity of the PbDtBn assembly (Tables 1 and 4). In total, it filled in 415 of 522 gaps (79.50%). As expected, gap-filling was able to fill far more small than large gaps, resulting in an increase of the gap N50 from 12,335 nt to 110,194 nt, a nearly 9-fold increase. The latter is much longer than typical PacBio reads and may represent repeats that were too long to span with these reads. The total gap length was only reduced by 8.82% despite the fact that 79.50% of the gaps were filled, again reflecting the preferential filling of small gaps. Nevertheless, continuity is much improved. The number of contigs dropped by ~12% to just over 1000 (1016 contigs), and the contig N50 increased from 3,768,504 nt to 5,925,378 nt, representing an increase of 57.23%. Gap filling had little effect on the number of scaffolds, scaffold N50, or total assembly size (differences between gap filled and ungapped assemblies were <0.5%.

The completeness stats of the gap filled assembly improved slightly relative to the PbDtBn assembly before gap-filling (Additional file 1: Tables S1 and S2). The final draft R108 v 1.0, assembly captured 93.2% of Pb reads and 96.8% of Illumina reads. Of the original Illumina readset, 95.8% were not only mapped but also properly paired, indicating that the assembly has captured most of the genome. The R108 v 1.p assembly has captured most of the gene space, with estimates ranging from 92.3% for the transcript assembly to 95.2% for the BUSCO assembly, and 55,706 genes predicted MAKER-P. Overall, this final draft of the R108 assembly captures nearly all the assembly and gene space.

### Novel sequences revealed by the R108 assembly

A new high quality reference sequence for R108 allowed a side-by-side comparison of two *Medicago* accessions (A17 and R108). We were able to build chromosome-level synteny blocks between R108 and A17. We also found extensive novel sequence in the R108 assembly that was not part of the A17 reference assembly (Table 5). There was nearly 23 Mb of R108 assembly sequence that could not be found in the A17 assembly. This represents 5.7% of the nucleotides in the R108 genome. These “novel” sequences are likely a mix of sequences that are truly novel in the R108 genome as well as sequences that are present in both genomes but have diverged beyond our ability to detect them or sequences that are in the A17 genome but didn’t make it into the A17 assembly. Out the nearly 23 Mb of novel R108 sequence, 1.6 Mb represent novel R108 coding sequence that could not be found in the A17 assembly, values quite similar to those observed with an earlier ALLPATHS-LG [48] assembly of R108 [39]. These regions contain candidate R108-specific genes or gene that were deleted from A17 or arose independently in the R108 lineage.

**Table 5:** R108 v 1.0 assembly characteristics in comparison to the A17 reference assembly

	Nucleotides	% Nucleotides
Total Bases	399,348,955	100.00%
Repetitive	96,760,262	24.23%
Alignable to A17	366,489,898	91.77%
Bases in Synteny with A17	283,853,354	71.08%
Novel Sequences vs A17	22,763,508	5.70%
Novel Coding Sequences vs A17	1,623,097	0.41%

### Chromosomal-scale translocation

**Fig. 1:**
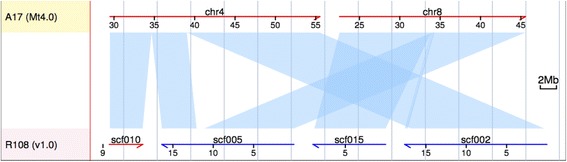
Synteny alignment of partial chromosomes 4 and 8 between A17 and R108 confirms rearrangement of the long arms of the chromosomes

Although R108 is phylogenetically distant from A17 compared to other accessions, we were able to align more than 280 Mb of syntenic regions in both genomes (Table 5), representing over 70% of the R108 assembly. These numbers also correspond well with sequence comparisons based on an earlier ALLPATHS-LG assembly of R108 [39]. Within these synteny blocks, extensive variations were discovered including single nucleotide changes, small insertions and deletions, as well as large structural changes such as inversion and translocation. While most structural changes were TE-related and only involve small local regions, we identified two large rearrangements on chromosomes 4 and 8 between R108 and A17. Through synteny comparison, we found one R108 scaffold (scf005, 16.4 Mb) spanning the upper arm of chromosome 4 and the lower arm of chromosome 8 in A17, and another two scaffolds (scf015, 12.0 Mb and scf002, 17.6 Mb) together spanning the upper arm of chromosome 8 plus the lower arm of chromosome 4 (Fig. 1), indicating a chromosomal-scale translocation between the reference *Medicago* accession (A17) and the widely-used R108 accession.

**Fig. 2:**
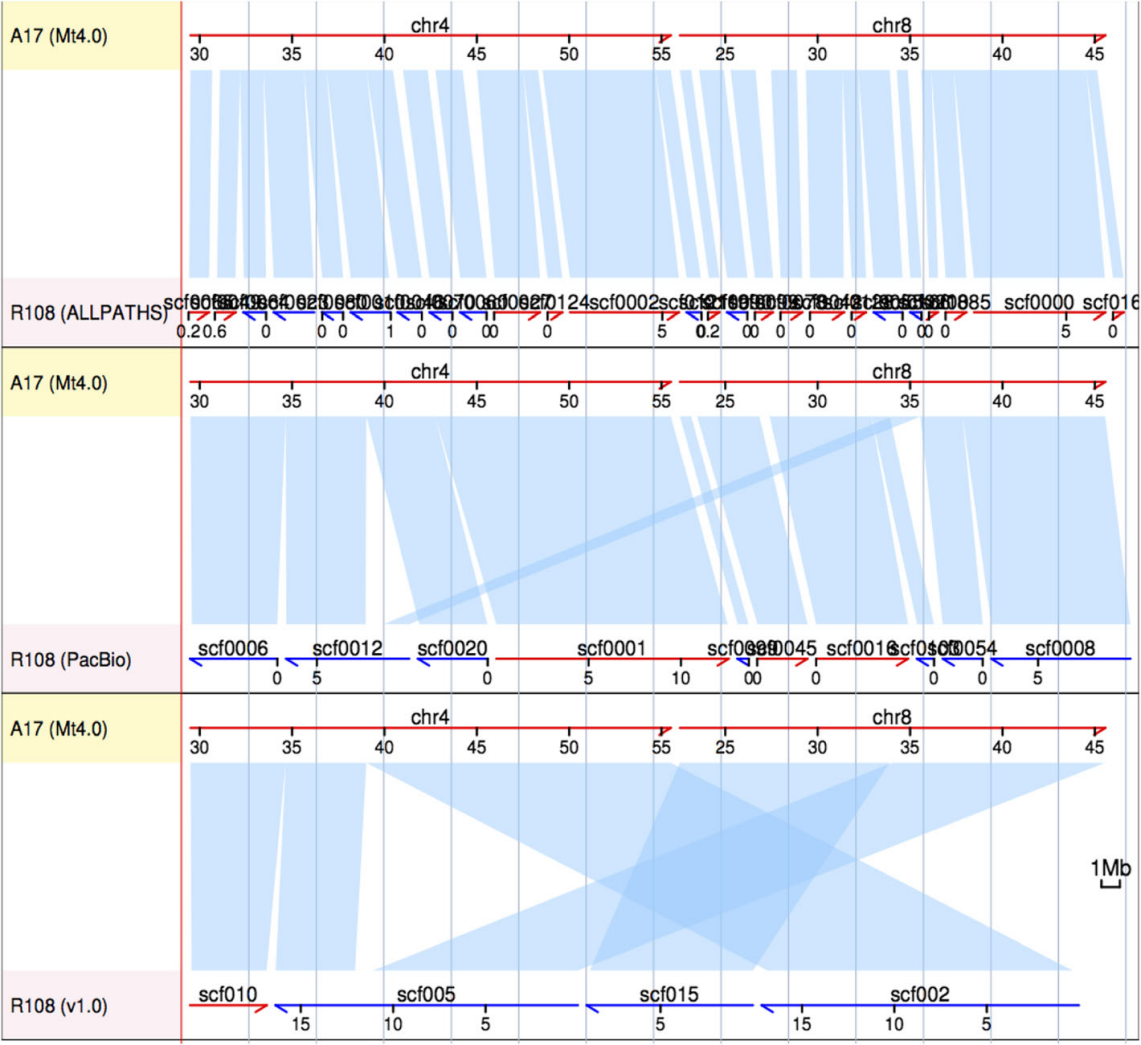
Synteny alignment of partial A17 chromosomes 4 and 8 against syntenic regions in the R108 Illumina-based assembly (*top panel*), PacBio-based assembly (Pb, *middle panel*) as well as the gap-filled PbDtBn (v1.0) assembly (*bottom panel*)

**Fig. 3:**
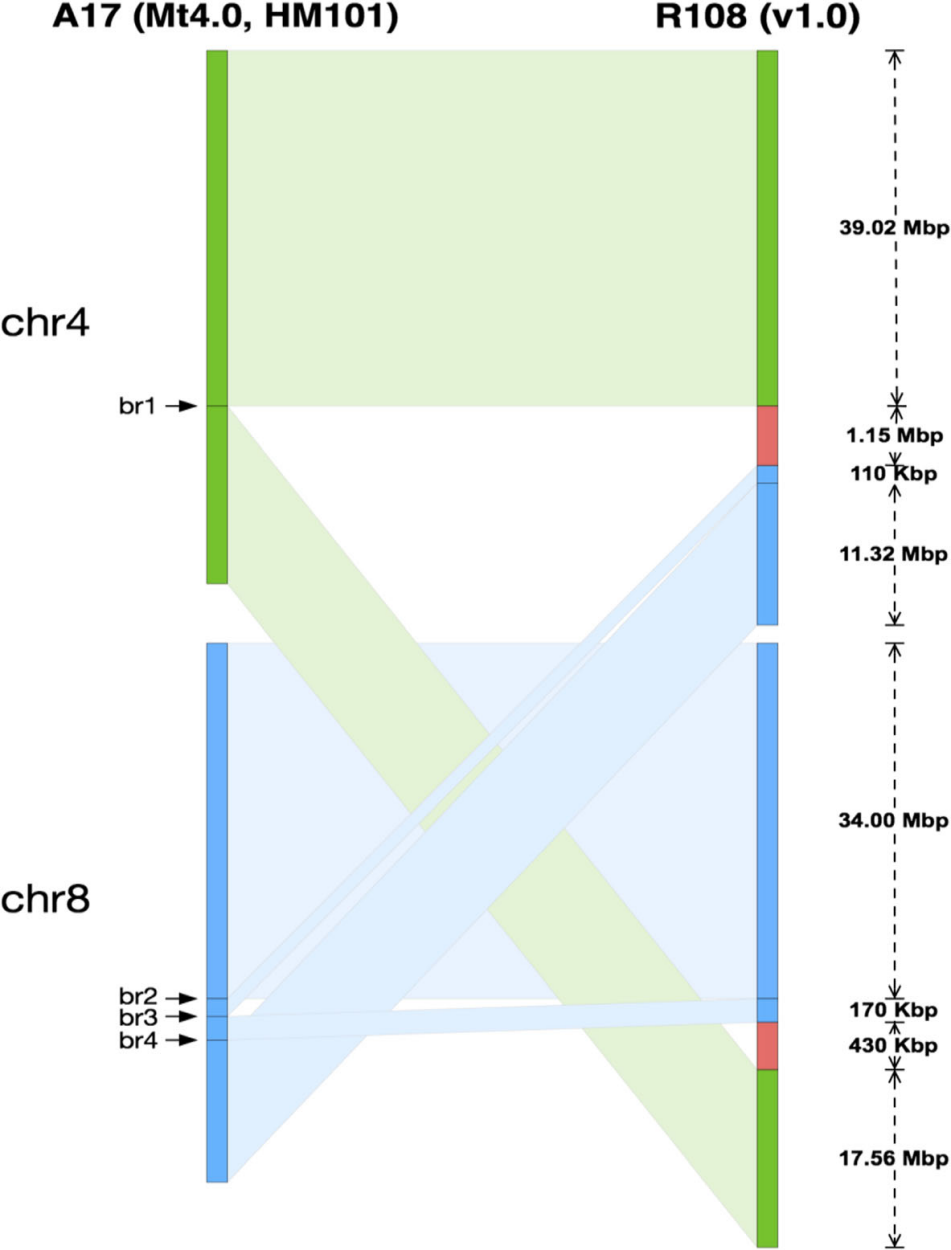
Schematic of the rearrangement between chromosomes 4 and 8 in A17 (*left*) compared to R108 (*right*). *Green* segments indicate homology to A17’s chromosome 4 while *blue* segments indicate homology to A17 chromosome 8. *Red* segments indicate sequences not present in the A17 reference). Breakpoint 1 (br1) is pinpointed to a 104 bp region (chr4:39,021,788-39,021,891) and includes a 100 bp gap. Breakpoint 2 (br2) is pinpointed to a 7665 bp region (chr8:33,996,308-34,003,972) and includes a 7663 bp gap. Breakpoint 3 (br3) is pinpointed to a 708 bp region (chr8: 34,107,285-34,107,992) and includes a 100 bp gap. Breakpoint 4 is pinpointed to a 277 bp region (chr8:34,275,249-34,275,525) and includes a 100 bp gap)

Previously, Kamphuis et al. reported a rearrangement between linkage groups 4 and 8 in the reference accession A17 relative to other accessions [49]. Using genetic markers and linkage mapping, the authors hypothesized a chromosomal-scale translocation private to A17 which involves the lower arms of chromosomes 4 and 8 [49]. To date, however, the physical location of the rearrangement has not been determined and, in fact, the rearrangement itself has not been elaborated through genome sequencing. Lack of high quality genome assemblies of non-A17 accessions certainly hindered such whole genome comparison. However, even with the whole genome assemblies available (including the earlier R108 ALLPATHS-LG assembly), it is still difficult to fully resolve rearrangement events at such chromosomal scale given the relatively short scaffold span of most sequencing and assembly techniques. Figure 2 clearly illustrates the improvements in resolving large-scale structural variation using long PacBio reads together with scaffolding or mapping technologies such as Dovetail and BioNano, over traditional Illumina-based assembly or assembly based on PacBio reads alone. Using the same synteny pipeline we aligned the Illumina-based R108 assembly, assembled with ALLPATHS-LG [48], to A17. The rearrangement region (~50 Mb) on chromosomes 4 and 8 was split into ~30 independent scaffolds in the ALLPATHS-LG R108 assembly (Fig. 2, top panel). The PacBio-based assembly (Pb), on the other hand, captured the region in ~10 scaffolds and partially resolved the breakpoint on chromosome 4 (Fig. 2, middle panel). With the aid of BioNano and Dovetail technologies, the affected region was captured in four long scaffolds in the final R108 assembly (PacBio + Dovetail + BioNano) with all breakpoints clearly resolved (Fig. 2, bottom panel). We were able to pinpoint exact breakpoints of the translocation to a single region on chromosome 4 and three regions on chromosome 8, something that could not be done with the Illumina-based ALLPATHS-LG assembly (Fig. 3). Interestingly, each of the four breakpoints involves a gap (i.e., ‘N’s) in the A17 reference, with one 7.5 kbp gap and three 100 bp gaps, the latter representing gaps of undetermined size (Haibao Tang, personal communication). These gaps indicate that the regions in and around the rearrangement breakpoints are structurally unstable, repetitive and/or difficult to assemble even using a BAC-by-BAC approach. We found numerous transposable element genes near the breakpoints, including a reverse transcriptase, a GAG-pre integrase and a cluster of 6 transferases near breakpoint 1, two helicases around breakpoint 2, two retrotransposons (UBN2) and two reverse transcriptases around breakpoint 3, and a MULE transposase right next to breakpoint 4. Intriguingly, a cluster of at least 10 CC-NBS-LRRs was found both upstream and downstream of breakpoint 2, and two CC-NBS-LRRs were also found right next to breakpoint 3, possibly suggesting a structural role of these resistance genes in plant genomes.

In addition to the translocation, we noticed two large stretches of R108 sequences (1.15 Mb and 430 Kb) downstream from the translocation breakpoints on chromosome 4 and 8 (Fig. 3 red segments) that didn’t have a syntenic match in A17. The chromosome 4 insertion in R108 is a ~ 1 Mb region with no synteny to A17 and right next to the chr4-8 translocation breakpoint. Both the translocation and insertion are found in several other accessions including HM034 and HM185 using a similar synteny comparison approach (data not shown). It is thus likely that the translocation is private to A17, which is consistent with [49], and this large insertion in R108 actually represents a private deletion in A17 which is expected to be found in the majority of *M. truncatula* accessions.

Further examination revealed that most of the insertion is novel. A total of 623 kbp of novel segments that do not align anywhere in A17 were identified in this region with 136 genes found in this region (Additional file 1: Table S5).

## Discussion

This work represents the first published example we are aware of examining multiple next generation scaffolding and mapping technologies in all possible combinations with a comparative analysis of their contributions. PacBio long reads combined with BioNano and Dovetail technologies have allowed us to generate a second, reference quality assembly for the model legume, *M. truncatula*, in the functionally-important R108 accession. In the process, we discovered important insights into how these technologies overlap and complement each other enabling us to propose an optimal strategy for their incorporation.

### Novel sequence was found in the R108 assembly

Long reads improve the continuity of assemblies [20, 50–54]. However, continuity is only one advantage of using long reads. The long reads help to correctly capture ambiguous regions of the genome in the assembly, including repeats and tandemly duplicated genes. Locally duplicated genes can be especially problematic as they are often collapsed or over-expanded in Illumina-only or even Illumina/PacBio hybrid assemblies (Miller et al., submitted). Using PacBio long reads, therefore, results in capture of additional sequence that is not possible with short reads. In addition, we capture accession specific sequences as well. In total, over 22 Mb of novel sequence, including 1.6 Mb of coding sequence were identified.

### Technologies made similar continuity gains and are valuable individually

Similar continuity gains were made by each technology in each round, as was seen in [6]. Both technologies improved the base Pb assembly, improving the 3.8 Mb scaffold N50 of the Pb assembly to just over 6.8 Mb (Table 1). Indeed, many of the same joins were made between both of the technologies. Both technologies, individually, were valuable in increasing continuity.

Despite the challenges of assembly the *M. truncatula* genome, with its history of whole genome duplication and high rate of locate duplication, there are many plant genomes that are much more complicated than the 500 Mb, largely homozygous *Medicago truncatula* genome. Increases in genome size, repetitive content, and the number of tandem, segmental, or whole genome duplications will change the dynamics of the assembly and the contributions of the technologies. In *Medicago* described here, the PacBio assembly came together quite well, making the improvements when using BioNano and Dovetail less dramatic than they might have been. As genome complexity increases, including repeat and duplication content, coherent PacBio assemblies become increasingly difficult. As PacBio assemblies become more fragmented with increased genome complexity, we expect that the improvement in the assembly when adding BioNano and/or Dovetail will become increasingly crucial, leading to greater relative improvements, even while becoming more challenging. The assembly improvement with both technologies should follow similar patterns with increased genome complexity until extremely high levels of complexity, especially repeat size, become limiting even for these technologies.

### Further gains were made using both technologies

Though similar gains were seen when using either scaffolding or mapping technology, the use of both technologies together increased continuity gains and join numbers further (Table 1 and Additional file 1: Table S3) [6]. With a combined approach the two technologies were complementary by enabling additional joins than either Dovetail or BioNano could make independently. Using both scaffolding technologies in either order (PbDtBn or PbBnDt) increased the scaffold N50 to just over 12.1 Mb (Table 1).

One explanation for the complementarity between the two technologies may be a function of the differences in biases of the two technologies. BioNano’s information content is in restriction sites and the distances between them. As such, BioNano is highly dependent on the motif density of the restriction enzymes used [55, 56], which can vary within a genome. Genomic regions where motif density is high become “fragile sites”, that destabilize the DNA and resulting in limited or no coverage in the maps, and breaks in the genome map contigs [5, 8, 16, 56]. In these regions scaffolding of the assembly simply cannot occur. By contrast, regions of the genome with too low of a density of cutting sites also will result in low label density and missed join opportunities (a minimum of eight restriction sites is required in each DNA molecule, which is a minimum of 150 kb).

Dovetail is based on Hi-C technology, an extension of chromosome conformation capture, which has its own documented biases [57, 58]. Dovetail’s information content is “contact probabilities,” indicating the probability that any two regions in the genome will be brought together during the ligation stage and is inversely correlated with distance. Dovetail, which incorporates Illumina sequencing, also inherits biases in next generation sequencing and alignment, such as biases in the amplification, shearing and mapping steps.

### Join accuracy appears to be higher in dovetail compared to BioNano

Using A17 synteny as a proxy for accuracy of joined R108 scaffolds, Dovetail had a much higher percentage of joins mapping to the same A17 chromosome compared to BioNano (93.75 vs 78.57%), suggesting that accuracy is higher in Dovetail than in BioNano. Further, when looking at joins in scaffolds supported by both technologies, Dovetail-supported BioNano joins mapped to the same A17 chromosome 90.91%, an increase of 12.34% over all BioNano joins. This suggests that Dovetail confirmation increases the accuracy of BioNano joins. BioNano-supported Dovetail joins, however, increased mapping to the same A17 chromosome by only 0.54%, suggesting that BioNano confirmation did little to improve Dovetail accuracy.

These data argue that Dovetail joins are more accurate than BioNano joins. However, we cannot rule out that the possibility that the larger distances that the BioNano technology spanned while joining scaffolds (described above) might make it less likely that two joined scaffolds fall into a region that is syntenic with A17 given that synteny tends to decrease with distance. BioNano-joined scaffolds, therefore, might map to multiple A17 chromosomes more than Dovetail-joined scaffolds due to synteny breakdown rather than inaccuracy of joins. However, given that BioNano gaps span less than 200 kb and that the majority of the R108 genome has synteny blocks with A17 that are greater than 1 Mb (Figs. 1, 2, 3) [39], we expect this different to be small and the difference between Dovetail and BioNano join accuracy to be real.

Alternatively, Dovetail breaks performed much worse than joins using A17 synteny as a measure. Of the PbBn scaffolds subsequently broken by Dovetail in the PbBnDt assembly, only 40% of them mapped to different A17 chromosomes, indicating that Dovetail might be breaking more correct BioNano joins than incorrect ones.

A17 chromosomal mapping is far from a perfect gold standard given the evolutionary distance between A17 and R108. Joined segments of R108 scaffolds that map to different A17 chromosomes may still map to the same R108 chromosome. Indeed, one of the joins shared by both Dovetail and BioNano that mapped to different A17 chromosomes corresponds to the known chromosome 4/8 translocation. This join, therefore, is correct, even though synteny to A17 put it on two different chromosomes. It is possible that there are other regions where synteny to A17 doesn’t accurately predict synteny in R108. Using long-range physical information, such as Hi-C data or a genetic map that involves R108, could allow us to better validate the BioNano and Dovetail technologies as well as to obtain chromosome-scale ordering of the genome assembly.

### Strengths and weaknesses dictate strategy for ordering technologies

For the final assembly, we chose to gap-fill the PbDtBn assembly rather than the PbBnDt assembly. This decision was based not only on comparisons of important assembly continuity and completeness statistics, as described above, but also on the knowledge we uncovered about the differences between the scaffolding and mapping technologies.

One important difference between the two technologies is their ability to incorporate smaller scaffolds. In our study, Dovetail incorporated thirty-five small scaffolds (less than 100 kb) over both rounds but BioNano incorporated only one. The minimum scaffold size joined by BioNano (98.1 kb) was more than 20 times larger than the minimum scaffold size joined by Dovetail (4.8 kb). Similar results were found when applying BioNano maps to the short arm of wheat chromosome 7D where the optimum size for incorporation by BioNano was 90 kb or higher [56] and sequences shorter than 30 kb could not anchored reliably. Given that the scaffold N50 was 3.7 Mb in the Pb assembly to which these technologies was added, the discrepancy between the two technologies in joining scaffolds less than 100 kb did not have as great an effect on our assemblies. However, if a much more fragmented assembly were used, we would expect Dovetail to perform much better than BioNano if only one scaffolding or mapping technology were used. If both technologies are used, applying Dovetail first to incorporate the smaller scaffolds and create a more contiguous substrate for BioNano to use makes sense and would be especially critical for highly fragmented assemblies.

A second difference in the two technologies also supports applying Dovetail prior to BioNano for combined strategies. Dovetail breaks sequences it identifies as chimeric as it runs the software. BioNano logs potential chimeric sequences, but does not induce breaks in the assembly without manual intervention. Hence, if BioNano is applied first, chimeric contigs may not yet be properly separated when the assembler’s master plan for scaffolding is being formed. Having a more accurate assembly up-front, as should occur when Dovetail is applied first, is always best before scaffolding assemblies.

Both technologies were able to bridge larger duplicated and/or repetitive regions than was PacBio, which requires multiple reads long enough to span an ambiguous region. With only 10 % of the sequenced nts in PacBio reads longer than 18,555 nt (N10), the ability of PacBio to span ambiguous regions is likely limited to a similar size, though longer reads will increase the size of the spannable repeats. Therefore, both mapping technologies can add value for spanning ambiguous regions that are beyond the reach of current PacBio capabilities. However, both technologies are limited in the size of gap they can span. Dovetail is limited by its longest pairs, which in this study, likely kept joins to around 100 kb or less, though without sized gaps it is difficult to figure out the true maximum. BioNano can join scaffolds over much larger gaps. The largest span made in this study created a gap of nearly 650 kb, though most joins spanned less than 100 kb (Table 3). Nevertheless, Dovetail and BioNano both were able to span ambiguous regions that were beyond PacBio’s current capability.

## Conclusions

The use and analysis of both BioNano and Dovetail technologies in all possible combinations is novel and yielded strategic information about how best to apply these strategies to PacBio. Both technologies were able to span repetitive regions that PacBio was unable to bridge. Using PacBio, followed by Dovetail and then BioNano, and then gap-filled with PBJelly, we have generated a second, reference quality assembly for *M. truncatula*. Because of the distance between R108 and the A17 reference as well as the inability to interbreed them to create a genetic map, having a second high quality *M. truncatula* reference has been a priority in the *Medicago truncatula* community. A second reference assembly has yielded novel sequence and will be an important resource for the R108 functional community to support gene-finding in the *Tnt1* lines. The R108 reference assembly has also allowed us to investigate the details of the A17 translocation.

## Methods

We generated five genome assemblies: a PacBio only assembly (Pb), a PacBio base assembly that was scaffold together with either Dovetail (PbDt) or BioNano (PtBn), a Pb base assembly that was scaffold together with Dovetail and then BioNano (PbDtBn) and a Pb base assembly that was scaffold together with BioNano and then Dovetail (PbBnDt). The completeness of each assembly was evaluated by alignments of PacBio reads as well as independent Illumina reads, and capture of an independent transcriptome as well as core eukaryotic genes. For comparison, we used the A17 version 4.0 reference genome [44].

### PacBio sequencing and assembly

DNA for PacBio assemblies was obtained from fifty grams of young leaf tissue obtained from multiple plants grown in the greenhouse and dark-treated for 24 h. High molecular weight genomic DNA was generated by Amplicon Express (Pullman, WA) using their standard BAC nuclei prep followed by a CTAB liquid DNA precipitation.

Whole-genome DNA sequencing was performed using a Pacific Biosciences RS II instrument (Pacific BioSciences, Menlo Park, CA). Libraries were constructed using the PacBio 20-Kb protocol [59]. These libraries were loaded onto 122 SMRT cells and sequenced using P4/P6 polymerase and C2/C4 chemistry with 3- and 6-h movie times, respectively. PacBio sequencing yielded approximately 107X sequence coverage. A de novo assembly of PacBio reads was generated using FALCON [20] assembler version 0.4 using default parameters. Contigs smaller than 1 kb were removed. In order to improve the accuracy of the assembly, Quiver polishing was done on SMRT portal (version smrtanalysis_2.3.0.140936.p5.167094) using the “RS_Resequencing” protocol using the latest version available at the time.

### Dovetail

DNA from Amplicon Express (described above) was used. A Chicago library (Dovetail Genomics LLC, Santa Cruz, CA) [18] was generated using the *DpnII* restriction endonuclease (GATC). Briefly, this entailed reconstituting chromatin using purified histones and chromatin assembly factors, followed by cross-linking the chromatin using formaldehyde. DNA was then digested using the *DpnII* restriction endonuclease. The resulting sticky ends were filled in with thiolated and biotinylated nucleotides. A blunt end ligation of free ends followed by removal of the crosslinking and proteins yielded fragments with DNA joined across distances of up to about 100 kb. An exonuclease was used to remove the biotinylated nucleotides. The thiolated nucleotides, which were proximal to the biotinylated nucleotides, protected the DNA from further exonucleation.

The resulting DNA fragments were taken through a standard Illumina library prep, including shearing and adapter ligation. The library was sequenced on an Illumina HiSeq 2000 (2 × 100 Base Pairs) to a physical coverage level of ~588X (67X sequence coverage).

Sequence data generated from this library were used to scaffold the PacBio de novo assembly through Dovetail’s HiRise™ pipeline v. 1.3.0-57-g4d1fc9b [18]. In short, Chicago library reads were mapped back to the assembly using a modified version of SNAP (http://snap.cs.berkeley.edu/). Pairs in which both reads were uniquely mapped were used to generate a likelihood model representing how chromatin crosslinking brings sequences together. A graph where the nodes are contigs and the edges are ordered integer pairs representing placement of the paired reads in the contigs was used for scaffolding beginning with high confidence linear subpaths and prioritizing joins in order of log likelihood improvement. During the process, in addition to joining sequences, putative chimeric sequences were broken. An iterative approach was taken by feeding the resulting scaffolds back into the pipeline. Refinement of local ordering and orientation and gap closing using Meraculous’s Marauder module was done at the end [60].

### BioNano

Five grams of young leaf tissue was obtained from greenhouse-grown plants dark-treated for 24 h before harvest. High molecular weight DNA was extracted and a de novo whole genome map assembly was generated using the BioNano Genomics (BNG) (BioNano Genomics, San Diego, CA) platform at the Bioinformatics Center at Kansas State University. High Molecular Weight (HMW) DNA was nicked and labeled according to the IrysPrep protocol. In brief, HMW DNA was double digested by a cocktail of single-stranded nicking endonucleases, *Nt.BspQI* (GCTCTTC) and *Nt.BbvCI* (CCTCAGC), and then labeled with a fluorescent-dUTP nucleotide analog using Taq polymerase. Nicks were ligated with Taq DNA ligase and the backbone of the labeled DNA was stained using the intercalating dye, YOYO-1. The nicked and labeled DNA was then loaded onto an IrysChip for imaging automatically on the Irys system (BioNano Genomics). BNG molecules were filtered with a minimum length of 150 kb and 8 minimum labels. A *p*-value threshold for the BNG assembler was set to a minimum of *2.6e-9*. Molecules were assembled with BioNano Pipeline Version 2884 and RefAligner Version 2816 [55].

For BioNano scaffolding, *hybridScaffold.pl* version 4618 from BioNano Genomics was used. The input assembly fasta sequence was nicked in silico for *Nt.BspQI* and *Nt.BbvCI* labels. Consensus Maps (CMAP) were only created for scaffolds >20 kbp with >5 labels. A *p*-value of *1e-10* was used as a minimum confidence value to output initial (BNG consensus map to in silico cmap). The final (in silico cmap to final hybrid cmap) alignments and a *p*-value of *1e-13* were used as minimum confidence value to flag chimeric/conflicting alignments and to merge alignments. Scaffolds that were not super-scaffolded were added to the output from *hybridScaffold.pl*.

The BNG scaffolding pipeline identifies potential breaks that should be made to the base assembly in the form of a chimera file, but these suggested breaks are not made without manual intervention. We did not attempt to make any of the BioNano breaks. For BioNano joins, only joins that incorporated more than one scaffold were considered.

BioNano sizes gaps but does not fill them exclusively with Ns. Rather, BioNano adds in restriction site recognition sequences within the gap according to where restriction sites were seen in the BioNano map. This results in hundreds of tiny contigs which break up the BioNano gaps into smaller fragments. For the purposes of this paper, we used the GAEMR basic stats default of using 200 as a minimum contig size, effectively ignoring these restriction sites island for calculating assembly statistics and obtaining a single gap per join.

### Illumina

In order to compare the completeness of assemblies constructed with different combinations of PacBio, Dovetail, and BioNano, we collected Illumina data that was independent of the assemblies. Illumina short-insert paired ends were generated from an independent DNA sample using TrueSeq v3.0 chemistry and sequenced on an Illumina HiSeq® 2000. A total of 332,236,248 reads (71.4X coverage) of length 100 nt were generated.

### Transcriptome assembly

To evaluate how the transcriptome was represented in the genome assemblies, the transcriptome of 14 day old R108 roots was sequenced using Illumina’s RNA-Seq protocol. The transcriptome was assembled using the Transcriptome Assembly Pipeline (BPA2.1.0) [61]. The BPA pipeline includes a kmer sweep assembly strategy with ABySS (using the kmer values of 50, 60, 70, 80 and 90) [62], followed by an OLC (overlap layout consensus) assembly with CAP3 [63] to find overlaps between contigs (unitigs). Scaffolding with ABySS and gap closure were performed to obtain the final assembled transcriptome sequences (Simpson et al. 2009). The transcripts were clustered at 98% sequence identity using the CD-HIT-EST software [64]. Finally, the set of transcript sequences were filtered by length (minimum length of 100 bp). An additional filtering step using ESTScan [65] was performed to identify open reading frames using *M. truncatula* protein coding genes as a reference, yielding the final transcriptome set. Transcripts were mapped against each of the five assemblies using GMAP [66]. Transcript hits were retained if aligning along at least 90% of their sequence with at least 90% identity.

### BUSCO

Benchmarking Universal Single Copy Orthologs (BUSCO) provides a quantitative assessment of genome assemblies based on orthologs selected from OrthoDB [47]. Assembly assessments were performed using the plant early release of BUSCO v1.1b1, which contains 956 genes that are present in at least 90% of the plant species used to assemble the database [47]. tBLASTn searches were used to identify BUSCOs followed by Augustus gene predictions and classified into lineage specific matches using HMMER within the BUSCO package.

### Read alignments

In order to assess the completeness of the assembly, PacBio filtered (minimum length of 50 and minimum quality of 75) subreads were realigned to the five assemblies using the BLASR mapper [67]. All the subreads were considered for the alignment to the assemblies (−useallccs). Illumina reads were aligned to the five assemblies using the Burrows-Wheeler Aligner (BWA), version 0.7.12 with a maximum of 2 paths and sam output format.

### Structural annotation

To understand how gene sequences were affected by the assembly strategies, the MAKER-P genome annotation pipeline was used to annotate the five genome assemblies [68–70]. All available *M. truncatula* R108 transcripts were assembled using the Trinity Assembler. All transcripts were from a single tissue, root, which is not ideal. Nevertheless, GMAP alignments to A17 indicate that the transcript assembly contains the majority of genes. Further, within the five assemblies, relative capture rates of these transcripts should not be biased by the lack of evidence transcripts from multiple tissues.

The resulting assembly was used as input for expressed sequence tag (EST) evidence for MAKER-P annotations [71, 72]. The MAKER-P pipeline aligns the provided ESTs to the genome and creates ab initio gene predictions with SNAP [73] and Augustus [74, 75] using evidence-based quality values. Each assembly was divided into ten chunks and processed through MAKER-P individually. Following completion of MAKER-P runs for each of the ten chunks, fasta and gff files were combined using fasta_merge and gff3_merge, respectively, included as part of the MAKER-P package.

### Identification of structural rearrangements and novel sequences in R108

Each R108 PacBio-based assembly was first aligned to the A17 reference (i.e., Mt4.0) using BLAT [76]. The resulting alignments were merged, fixed (removing non-syntenic or overlapping alignment blocks) and cleaned (removing alignment blocks containing assembly gaps). BLAT Chain/Net tools were then used to obtain a single coverage best alignment net in the target genome (HM101) as well as a reciprocal-best alignment net between genomes. Finally, genome-wide synteny blocks were built for each assembly (against HM101), enabling identification of genome structural rearrangements including the chr4-8 translocation.

Based on pairwise genome comparison of R108 and A17, we obtained a raw set of novel sequences (present in R108 but absent in A17) by subtracting all aligned regions from the gap-removed assembly. Low-complexity sequences and short tandem repeats were scanned and removed using Dustmasker [77] and Tandem Repeat Finder [78]. Potential contaminant sequences (best hit in non-plant species) were filtered by BLASTing [79] against NCBI Nucleotide (nr/nt) database. Genes with more than 50% CDS in these regions comprised the accession-specific gene set. Pfam analysis and functional enrichment were then performed on this novel gene list [80].

## Additional files

Additional file1: Supplementary Figures and Tables. Contains Supplementary Figure S1 and Supplementary Tables S1-S5. (DOCX 110 kb)

## Abbreviations

Bn: BioNano
Dt: Dovetail
Pb: PacBio
PbBn: PacBio BioNano
PbBnDt: PacBio BioNano Dovetail
PbDt: PacBio Dovetail
PbDtBn: PacBio Dovetail BioNano
