# Supplementary material for: Strategies for optimizing BioNano and Dovetail explored through a second reference quality assembly for the legume model, Medicago truncatula
Source: BMC Genomics. 2017 Aug 4;18:578. doi: 10.1186/s12864-017-3971-4 (PMC5545040; doi:10.1186/s12864-017-3971-4)
Supplement: Supplementary file 1 — Supplementary Figures and Tables. Contains Supplementary Figure S1 and Supplementary Tables S1-S5. (DOCX 110 kb) [file 12864_2017_3971_MOESM1_ESM.docx]

**Supplementary Figures and Tables**


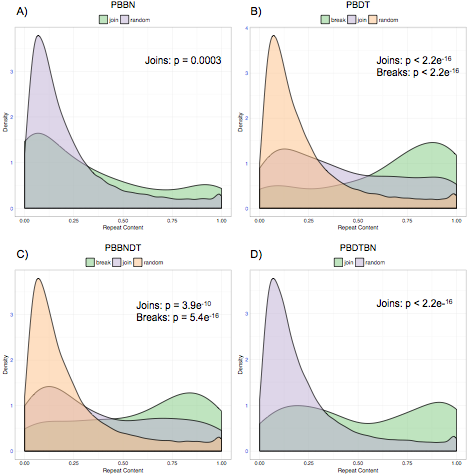


**Figure S1.** Density distribution of the repetitive content (0-100%) in 10kb flanking regions of “Joins” or “Breaks” made by BioNano (A) and (D) or Dovetail (B) and (C) as well as randomly selected 10kb regions across the entire assembly. (A) PbBn assembly. (B) PbDt assembly. (C) PbBnDt assembly. (D) PbDtBn assembly. Independent 2-group t-test p-values are shown comparing the repetitive content in (a) 10kb flanking regions of “Joins” or “Breaks” versus (b) randomly selected 10kb regions across the entire assembly, with the alternative hypothesis of (a) is greater than (b). Significance (p ≤ 0.05) indicates that the joins or breaks had significantly higher repetitive content than similar segments randomly chosen across the genome.

**Table S1. Assembly Completeness Estimated by Read Capture**

| Assembly | PacBio Reads^*^ Captured (%) | Illumina Reads^**^ Captured (%) | Illumina Reads^**^ Captured and Properly Paired (%) |
| --- | --- | --- | --- |
| Pb | 6,594,288 (91.76%) | 321,866,712 (96.78%) | 318,399,275 (95.74%) |
| PbBn | 6,594,243 (91.75%) | 321,866,643 (96.78%) | 318,399,409 (95.74%) |
| PbDt | 6,594,220 (91.76%) | 321,8671,46 (96.78%) | 318,399,550 (95.74%) |
| PbBnDt | 6,594,327 (91.76%) | 321,866,893 (96.78%) | 318,399,662 (95.74%) |
| PbDtBn | 6,594,166 (91.75%) | 321,867,071 (96.78%) | 318,402,805 (95.74%) |
| R108 v 1.0 | 6,699,888 (93.22%) | 321,938,947 (96.81%) | 318,346,398 (95.82%) |

^*^ Out of 7,186,829 PacBio reads

^**^ Out of 332,236,248 Illumina reads

**Table S2. Gene Space Completeness Estimated by Gene Capture and Annotation.**

| Assembly | BUSCO Capture^*^: Complete (%) /  Duplicated (%) | Transcriptome Capture^**^: Complete (%) /  Duplicated (%) | Maker gene annotations |
| --- | --- | --- | --- |
| Pb | 878 (91.84%) /  151 (15.79%) | 94,519 (92.24%) /  45,882 (44.78%) | 54,111 |
| PbBn | 881 (92.15%) /  152 (15.90%) | 94,529 (92.25%) /  45,868 (44.76%) | 55,494 |
| PbDt | 909 (95.08%) /  157 (16.42%) | 94,567 (92.29%) /  45,865 (44.76%) | 54,918 |
| PbBnDt | 913 (95.50%) /  154 (16.11%) | 94,555 (92.27%) /  45,873 (44.77%) | 55,921 |
| PbDtBn | 913 (95.50%) /  157 (16.42%) | 94,528 (92.25%) /  45,833 (44.73%) | 55,477 |
| R108 v 1.0 | 910 (95.19%) / 156 (16.32%) | 94,559 (92.28%) / 45,843 (44.74%) | 55,706 |

^*^ Out of 956 universal plant single copy ortholog groups.

^**^ Out of 102,472 transcripts.

**Table S3.** Joins made by BioNano and Dovetail.

|  | Total number of scaffolds joined | Average number of scaffolds joined | Resulting Scaffolds |
| --- | --- | --- | --- |
| BioNano_to_Pb | 140 | 2.75 | 50 |
| BioNano_to_PbDt | 96 | 2.91 | 33 |
| Dovetail_to_Pb | 172 | 2.64 | 64 |
| Dovetail_to_PbBn | 114 | 2.44 | 45 |

**Table S4.** Lengths of scaffolds joined with addition of Dovetail or BioNano.

| Addition of Dovetail  Pb -> PbDt | Addition of BioNano  Pb -> PbBn | Addition of BioNano  PbDt -> PbDtBn | Addition of Dovetail  PbBn -> PbBnDt |
| --- | --- | --- | --- |
| 4,765 | 172,295 | 98,093 | 4,765 |
| 13,859 | 184,107 | 107,710 | 12,305 |
| 17,365 | 191,416 | 111,413 | 13,859 |
| 19,528 | 197,064 | 117,154 | 17,365 |
| 20,462 | 199,197 | 119,117 | 19,528 |
| 24,606 | 204,792 | 139,332 | 24,606 |
| 40,328 | 211,397 | 147,814 | 40,328 |
| 40,603 | 221,942 | 165,597 | 40,603 |
| 45,421 | 222,674 | 181,029 | 45,421 |
| 46,452 | 229,787 | 184,193 | 46,452 |
| 50,449 | 236,881 | 191,416 | 50,449 |
| 72,062 | 242,526 | 198,420 | 72,062 |
| 74,559 | 246,008 | 198,748 | 72,105 |
| 87,608 | 261,179 | 199,197 | 74,559 |
| 95,516 | 263,253 | 204,792 | 87,608 |
| 96,308 | 286,128 | 221,942 | 95,516 |
| 97,822 | 312,830 | 236,881 | 96,308 |
| 100,498 | 317,082 | 242,526 | 97,822 |
| 112,143 | 333,145 | 294,948 | 100,498 |
| 114,044 | 337,409 | 311,565 | 105,178 |
| 116,701 | 342,213 | 312,830 | 112,143 |
| 119,211 | 342,956 | 317,082 | 114,044 |
| 123,877 | 351,632 | 333,145 | 116,701 |
| 132,910 | 352,385 | 333,496 | 119,211 |
| 136,534 | 356,256 | 419,138 | 123,877 |
| 138,318 | 369,708 | 434,464 | 124,895 |
| 141,368 | 384,623 | 456,256 | 136,534 |
| 144,778 | 390,302 | 461,133 | 138,318 |
| 172,295 | 441,626 | 537,759 | 141,368 |
| 184,107 | 452,217 | 545,815 | 144,910 |
| 197,064 | 495,267 | 567,577 | 184,107 |
| 221,400 | 497,056 | 570,760 | 221,400 |
| 222,674 | 544,336 | 582,932 | 262,376 |
| 240,718 | 554,331 | 587,485 | 276,383 |
| 246,008 | 561,919 | 592,369 | 295,454 |
| 261,179 | 570,760 | 604,500 | 309,549 |
| 262,376 | 578,942 | 613,619 | 324,954 |
| 263,253 | 582,932 | 638,510 | 348,044 |
| 276,383 | 587,485 | 668,813 | 379,584 |
| 286,128 | 604,500 | 753,599 | 383,370 |
| 295,454 | 713,271 | 794,226 | 406,393 |
| 309,549 | 733,860 | 808,820 | 543,268 |
| 324,954 | 733,870 | 845,913 | 561,919 |
| 337,409 | 748,912 | 930,745 | 566,884 |
| 342,213 | 753,599 | 939,813 | 622,052 |
| 342,956 | 753,867 | 948,911 | 654,116 |
| 348,044 | 759,290 | 985,622 | 713,271 |
| 351,632 | 764,384 | 1,026,723 | 740,172 |
| 352,385 | 778,032 | 1,097,352 | 773,553 |
| 356,256 | 781,648 | 1,193,346 | 791,712 |
| 369,708 | 794,226 | 1,349,103 | 800,847 |
| 379,584 | 823,006 | 1,425,957 | 823,425 |
| 383,370 | 825,362 | 1,516,424 | 841,688 |
| 384,623 | 836,349 | 1,520,651 | 994,715 |
| 390,302 | 842,479 | 1,587,132 | 1,058,110 |
| 406,393 | 854,372 | 1,599,774 | 1,068,876 |
| 441,626 | 862,174 | 1,676,003 | 1,100,880 |
| 452,217 | 880,303 | 1,781,200 | 1,105,872 |
| 466,679 | 929,179 | 1,797,763 | 1,111,575 |
| 495,267 | 933,973 | 1,872,056 | 1,165,719 |
| 497,056 | 935,073 | 1,908,307 | 1,278,202 |
| 543,268 | 940,558 | 1,985,506 | 1,338,792 |
| 554,331 | 948,911 | 2,064,453 | 1,339,347 |
| 561,919 | 1,009,576 | 2,299,207 | 1,356,297 |
| 566,884 | 1,021,810 | 2,482,584 | 1,387,510 |
| 578,942 | 1,039,454 | 2,702,969 | 1,427,073 |
| 622,052 | 1,042,934 | 2,807,741 | 1,448,806 |
| 654,116 | 1,056,227 | 2,917,016 | 1,527,803 |
| 713,271 | 1,059,083 | 3,043,675 | 1,629,415 |
| 733,860 | 1,073,967 | 3,198,926 | 1,717,152 |
| 733,870 | 1,097,352 | 3,333,935 | 1,778,365 |
| 740,172 | 1,111,575 | 3,360,667 | 1,895,823 |
| 748,912 | 1,183,273 | 3,365,678 | 1,934,573 |
| 753,867 | 1,189,477 | 3,515,994 | 1,970,468 |
| 759,290 | 1,207,010 | 3,599,395 | 2,112,952 |
| 764,384 | 1,212,855 | 3,872,939 | 2,239,460 |
| 773,553 | 1,217,324 | 3,990,443 | 2,358,872 |
| 778,032 | 1,250,294 | 4,007,054 | 2,428,748 |
| 781,648 | 1,332,612 | 4,154,966 | 2,467,599 |
| 800,847 | 1,338,792 | 4,314,535 | 2,538,677 |
| 823,425 | 1,514,642 | 4,589,331 | 2,677,381 |
| 830,449 | 1,527,803 | 4,684,924 | 2,745,343 |
| 836,349 | 1,560,574 | 5,395,695 | 2,788,049 |
| 841,688 | 1,599,774 | 6,136,307 | 2,936,575 |
| 842,479 | 1,676,003 | 6,348,449 | 3,495,296 |
| 854,372 | 1,690,670 | 6,895,511 | 3,536,248 |
| 862,174 | 1,708,090 | 7,916,921 | 3,597,609 |
| 878,204 | 1,727,240 | 8,431,636 | 3,667,709 |
| 929,179 | 1,785,465 | 10,609,486 | 3,800,436 |
| 933,973 | 1,859,904 | 10,740,307 | 3,851,214 |
| 935,073 | 1,872,056 | 10,822,442 | 3,977,283 |
| 940,558 | 1,911,723 | 11,191,479 | 4,103,722 |
| 994,715 | 2,050,451 | 11,414,498 | 4,215,660 |
| 1,009,576 | 2,064,453 | 16,842,575 | 4,223,009 |
| 1,021,810 | 2,086,283 | 17,052,781 | 4,732,641 |
| 1,039,454 | 2,150,489 | 19,275,758 | 4,855,511 |
| 1,042,934 | 2,161,911 |  | 4,980,734 |
| 1,056,227 | 2,165,192 |  | 5,197,047 |
| 1,059,083 | 2,168,296 |  | 5,685,865 |
| 1,068,876 | 2,259,868 |  | 5,706,248 |
| 1,073,967 | 2,263,624 |  | 5,807,674 |
| 1,111,575 | 2,473,936 |  | 6,819,834 |
| 1,183,273 | 2,482,584 |  | 8,053,651 |
| 1,189,477 | 2,508,716 |  | 8,190,324 |
| 1,207,010 | 2,524,296 |  | 8,293,303 |
| 1,212,855 | 2,677,381 |  | 8,811,391 |
| 1,217,324 | 2,721,202 |  | 9,372,885 |
| 1,250,294 | 2,746,058 |  | 9,584,062 |
| 1,332,612 | 2,807,711 |  | 9,640,055 |
| 1,338,792 | 2,820,044 |  | 10,145,405 |
| 1,356,297 | 2,832,725 |  | 12,056,195 |
| 1,387,510 | 2,958,125 |  | 13,613,478 |
| 1,427,073 | 3,009,654 |  | 17,638,354 |
| 1,448,806 | 3,029,716 |  | 22,885,216 |
| 1,514,642 | 3,198,926 |  |  |
| 1,527,803 | 3,248,877 |  |  |
| 1,560,574 | 3,301,056 |  |  |
| 1,629,415 | 3,354,324 |  |  |
| 1,690,670 | 3,360,667 |  |  |
| 1,727,240 | 3,698,567 |  |  |
| 1,778,365 | 3,768,504 |  |  |
| 1,859,904 | 3,939,538 |  |  |
| 1,911,723 | 3,957,684 |  |  |
| 1,934,573 | 4,007,054 |  |  |
| 2,050,451 | 4,314,535 |  |  |
| 2,086,283 | 4,450,890 |  |  |
| 2,150,489 | 4,463,762 |  |  |
| 2,161,911 | 5,395,695 |  |  |
| 2,165,192 | 6,674,656 |  |  |
| 2,168,296 | 6,799,422 |  |  |
| 2,259,868 | 6,961,591 |  |  |
| 2,263,624 | 7,400,057 |  |  |
| 2,473,936 | 7,532,372 |  |  |
| 2,508,716 | 8,076,284 |  |  |
| 2,524,296 | 8,125,060 |  |  |
| 2,677,381 | 9,426,113 |  |  |
| 2,721,202 | 11,414,498 |  |  |
| 2,746,058 | 12,056,195 |  |  |
| 2,807,711 | 12,588,919 |  |  |
| 2,832,725 | 13,488,151 |  |  |
| 2,936,575 |  |  |  |
| 2,958,125 |  |  |  |
| 3,009,654 |  |  |  |
| 3,029,716 |  |  |  |
| 3,248,877 |  |  |  |
| 3,301,056 |  |  |  |
| 3,354,324 |  |  |  |
| 3,768,504 |  |  |  |
| 3,851,214 |  |  |  |
| 3,939,538 |  |  |  |
| 3,957,684 |  |  |  |
| 3,977,283 |  |  |  |
| 4,103,722 |  |  |  |
| 4,450,890 |  |  |  |
| 4,463,762 |  |  |  |
| 4,470,962 |  |  |  |
| 4,855,511 |  |  |  |
| 4,980,734 |  |  |  |
| 5,685,865 |  |  |  |
| 6,033,237 |  |  |  |
| 6,674,656 |  |  |  |
| 6,799,422 |  |  |  |
| 6,961,591 |  |  |  |
| 7,400,057 |  |  |  |
| 7,532,372 |  |  |  |
| 8,076,284 |  |  |  |
| 8,125,060 |  |  |  |
| 9,372,885 |  |  |  |
| 9,426,113 |  |  |  |
| 12,056,195 |  |  |  |
| 12,588,919 |  |  |  |
| 13,488,151 |  |  |  |

**Table S5.** Novel R108 genes found near the chromosome 4/8 translocation.

| scaffold | start | end | Pfam_category |
| --- | --- | --- | --- |
| scf005 | 11095641 | 11095949 | NA |
| scf005 | 11118227 | 11118493 | NA |
| scf005 | 11119543 | 11123645 | Alg6_Alg8 |
| scf005 | 11119583 | 11119771 | NA |
| scf005 | 11127569 | 11127892 | Glutaredoxin |
| scf005 | 11127602 | 11127901 | NA |
| scf005 | 11135222 | 11135644 | Sugar_tr |
| scf005 | 11137726 | 11139732 | Sugar_tr |
| scf005 | 11149222 | 11149830 | Sugar_tr |
| scf005 | 11152556 | 11155450 | Sugar_tr |
| scf005 | 11162375 | 11171730 | B3_4 |
| scf005 | 11173902 | 11175118 | Str_synth |
| scf005 | 11191348 | 11192593 | NA |
| scf005 | 11192746 | 11194194 | TCP |
| scf005 | 11201024 | 11205737 | Aminotran_3 |
| scf005 | 11211809 | 11216831 | KH_1 |
| scf005 | 11226831 | 11227178 | NA |
| scf005 | 11229069 | 11229194 | NA |
| scf005 | 11229071 | 11229677 | FMN_dh |
| scf005 | 11230417 | 11231011 | FMN_dh |
| scf005 | 11231563 | 11232310 | FMN_dh |
| scf005 | 11233353 | 11235245 | PPR_2 |
| scf005 | 11239644 | 11240317 | FMN_dh |
| scf005 | 11241597 | 11243518 | FMN_dh |
| scf005 | 11244237 | 11246228 | FMN_dh |
| scf005 | 11257371 | 11259345 | FMN_dh |
| scf005 | 11261967 | 11265415 | FMN_dh |
| scf005 | 11266328 | 11266510 | NA |
| scf005 | 11267879 | 11270080 | FMN_dh |
| scf005 | 11280311 | 11282861 | CCT_2 |
| scf005 | 11320154 | 11320558 | NA |
| scf005 | 11327187 | 11328308 | NA |
| scf005 | 11346749 | 11348350 | GRAS |
| scf005 | 11349822 | 11349980 | RepeatMasker |
| scf005 | 11360307 | 11363761 | Ssu72 |
| scf005 | 11365793 | 11368088 | Thioredoxin_4 |
| scf005 | 11374930 | 11376645 | DUF3317 |
| scf005 | 11377407 | 11382200 | UQ_con |
| scf005 | 11405892 | 11406962 | NdhL |
| scf005 | 11442902 | 11443075 | NA |
| scf005 | 11445913 | 11448857 | DUF26 |
| scf005 | 11455102 | 11456094 | UQ_con |
| scf005 | 11464350 | 11465966 | NA |
| scf005 | 11469756 | 11470730 | UQ_con |
| scf005 | 11476641 | 11477621 | UQ_con |
| scf005 | 11493366 | 11496049 | MR_MLE_C |
| scf005 | 11497812 | 11500435 | MRP-S28 |
| scf005 | 11502159 | 11511565 | DUF789 |
| scf005 | 11538061 | 11539968 | zf-C2H2_6 |
| scf005 | 11551497 | 11551953 | NA |
| scf005 | 11555481 | 11559015 | DUF1423 |
| scf005 | 11562733 | 11564434 | Cytochrom_B561 |
| scf005 | 11585067 | 11587415 | Glyco_hydro_16 |
| scf005 | 11592149 | 11596063 | 2OG-FeII_Oxy_4 |
| scf005 | 11600813 | 11600926 | NA |
| scf005 | 11606503 | 11606937 | Ribosomal_S17e |
| scf005 | 11611188 | 11611568 | Ribosomal_S17e |
| scf005 | 11620516 | 11620770 | NA |
| scf005 | 11623594 | 11625456 | Metallophos |
| scf005 | 11627545 | 11627727 | NA |
| scf005 | 11628084 | 11629971 | Metallophos |
| scf005 | 11635030 | 11636590 | polyprenyl_synt |
| scf005 | 11642150 | 11643351 | Metallophos |
| scf005 | 11653591 | 11653833 | EF-hand_7 |
| scf005 | 11657769 | 11658524 | DYW_deaminase |
| scf005 | 11672553 | 11674524 | DIOX_N |
| scf005 | 11678795 | 11681301 | DIOX_N |
| scf005 | 11688189 | 11690041 | DIOX_N |
| scf005 | 11692025 | 11695686 | DIOX_N |
| scf005 | 11705181 | 11705902 | 2OG-FeII_Oxy |
| scf005 | 11707657 | 11707995 | DIOX_N |
| scf005 | 11708198 | 11709929 | 2OG-FeII_Oxy |
| scf005 | 11718512 | 11724722 | Zip |
| scf005 | 11725587 | 11726724 | NA |
| scf005 | 11732246 | 11733451 | ANTH |
| scf005 | 11734905 | 11736744 | GAGA_bind |
| scf005 | 11750701 | 11753272 | zf-C2H2 |
| scf005 | 11773647 | 11778842 | EMP70 |
| scf005 | 11781557 | 11781727 | Ribosomal_L14 |
| scf005 | 11781850 | 11784072 | NA |
| scf005 | 11834642 | 11838696 | K_trans |
| scf005 | 11858109 | 11859629 | NA |
| scf005 | 11863081 | 11865542 | SET |
| scf005 | 11868143 | 11868778 | PTR2 |
| scf005 | 11869790 | 11870602 | MFS_1 |
| scf005 | 11873919 | 11877524 | PTR2 |
| scf005 | 11879768 | 11879974 | B3 |
| scf005 | 11880687 | 11881014 | B3 |
| scf005 | 11882780 | 11886281 | PTR2 |
| scf005 | 11887627 | 11890364 | PTR2 |
| scf005 | 11898457 | 11903150 | PTR2 |
| scf005 | 11907263 | 11911149 | FAR1 |
| scf005 | 11913450 | 11914364 | NA |
| scf005 | 11918899 | 11921037 | Pyridoxal_deC |
| scf005 | 11924181 | 11927098 | PTR2 |
| scf005 | 11933886 | 11938515 | YL1 |
| scf005 | 11950353 | 11953515 | zf-CCCH |
| scf005 | 11956578 | 11956772 | NA |
| scf005 | 11972438 | 11975375 | PPR_2 |
| scf005 | 11977683 | 11981267 | zinc_ribbon_2 |
| scf005 | 11984127 | 11986976 | ATP-synt_F |
| scf005 | 11991647 | 11992549 | TPR_19 |
| scf005 | 11997224 | 11998000 | AAA_11 |
| scf005 | 11998069 | 11999227 | AAA_12 |
| scf005 | 11998500 | 11998712 | NA |
| scf005 | 11999741 | 12000003 | NA |
| scf005 | 12000001 | 12008266 | UvrD-helicase |
| scf005 | 12010877 | 12013236 | NA |
| scf005 | 12048648 | 12048908 | NA |
| scf005 | 12051487 | 12053450 | SNAP |
| scf005 | 12053652 | 12054866 | UvrD_C |
| scf005 | 12054976 | 12057560 | AAA_12 |
| scf005 | 12063176 | 12063933 | NA |
| scf005 | 11157462 | 11160525 | Sugar_tr |
| scf005 | 11221022 | 11225775 | DS |
| scf005 | 11288075 | 11291689 | Transket_pyr |
| scf005 | 11303832 | 11305658 | RPE65 |
| scf005 | 11413522 | 11420444 | ANTH |
| scf005 | 11433863 | 11437430 | Zein-binding |
| scf005 | 11484222 | 11485668 | Reticulon |
| scf005 | 11516411 | 11519515 | LRK10L-1 |
| scf005 | 11613298 | 11619479 | Metallophos |
| scf005 | 11647465 | 11649919 | Exo_endo_phos |
| scf005 | 11713177 | 11715601 | 2OG-FeII_Oxy |
| scf005 | 11762298 | 11765714 | LRR_X |
| scf005 | 11806588 | 11823309 | Na_H_Exchanger |
| scf005 | 11845172 | 11845654 | NA |
| scf005 | 11893654 | 11895825 | PTR2 |
| scf005 | 11942947 | 11946722 | DUF1084 |
| scf005 | 11958540 | 11963107 | Remorin_C |
| scf005 | 12019519 | 12030472 | AAA_12 |
| scf005 | 12038768 | 12046994 | RNA_pol_Rpb2_6 |
| scf005 | 11272043 | 11275429 | FMN_dh |
| scf005 | 11399264 | 11403946 | DUF2359 |
| scf005 | 11787045 | 11801809 | Na_H_Exchanger |
| scf005 | 12058085 | 12059543 | AAA_11 |
